# Diagnostic Accuracy of Artificial Intelligence Models for Differentiation of Squamous Cell Carcinoma and Adenocarcinoma of Lung—A Systematic Review

**DOI:** 10.3390/diagnostics16030500

**Published:** 2026-02-06

**Authors:** Kaushik Nayak, Rajagopal Kadavigere, Saikiran Pendem, Pallavi R. Mane, Niranjana Sampathila, Priya Pattath Sankaran, Nandish Siddeshappa

**Affiliations:** 1 Department of Medical Imaging Technology, Manipal College of Health Professions, Manipal Academy of Higher Education, Manipal, India; nayak.kaushik@manipal.edu; 2 Department of Radiodiagnosis and Imaging, Kasturba Medical College, Manipal Academy of Higher Education, Manipal, India; priya.ps@manipal.edu; 3 Manipal Institute of Technology, Manipal Academy of Higher Education, Manipal, India; palvi.mane@manipal.edu (P.R.M.); niranjana.s@manipal.edu (N.S.); 4 AI and Data Analytics Visukhi Innotech, Bangalore, India; nandnama@gmail.com

**Keywords:** artificial intelligence, Non-Small Cell Lung Carcinoma, computed tomography, squamous cell carcinoma, adenocarcinoma

## Abstract

**Background/Objectives**: Lung cancer remains the leading cause of cancer-related deaths worldwide, with Non-Small Cell Lung Cancer (NSCLC) accounting for the majority of cases, primarily Squamous Cell Carcinoma (SCC) and Adenocarcinoma (ADC). The aim of this systematic review is to summarise and critically appraise the performance of machine learning (ML)-based radiomics models in the differential diagnosis and overall survival analysis for lung SCC and ADC. **Methods:** PRISMA standards were followed in conducting the review. The quality of the studies was assessed using the Radiomics quality score (RQS) tool. **Results**: A total of 11 studies were included, demonstrating that deep learning and radiomics-based machine learning models significantly improve the non-invasive classification of lung squamous cell carcinoma and adenocarcinoma. Deep learning systems achieved an accuracy of 67–97%, and machine learning models showed an accuracy of 75–87%. The integration of radiomic features further enhanced diagnostic precision, showing strong potential for reliable histologic subtype differentiation. **Conclusions**: Machine learning-based radiomics models and deep learning significantly enhance the non-invasive, accurate differentiation of lung squamous and adenocarcinoma cell carcinoma when combined with clinical and pathological data.

## 1. Introduction

Lung cancer remains the foremost cause of cancer-related mortality worldwide. Non-Small Cell Lung Cancer (NSCLC) constitutes approximately 80% to 85% of all lung cancer cases, with Squamous Cell Carcinoma and Adenocarcinoma (ADC) representing the two most prevalent histological subtypes [1]. ADC is now the most common subtype, followed closely by SCC [2]. The early and accurate identification of these specific types is critical for initiating effective patient management. The increased credibility of advancements in imaging technology, particularly high-resolution computed tomography (CT), is a key factor in the improved early diagnosis of tumours in recent decades, allowing for better overall survival outcomes [3].

Accurate differentiation between lung SCC and ADC is mandatory, as these subtypes exhibit distinct biological behaviours, responses to targeted molecular therapies, and prognostic outcomes [4,5]. While histopathological biopsy remains the gold standard for definitive classification, it is an invasive procedure that may be subject to sampling bias and requires time, which can delay necessary systemic treatment [6]. Furthermore, studies indicate that SCC, often characterised by a more central location and higher rates of cavitation, generally confers a worse prognosis and higher recurrence risk than ADC, even when matched for stage [7,8,9]. These clinical realities underscore an urgent need for robust, non-invasive methods that can provide precise preoperative histological typing.

Radiomics incorporates a range of advanced quantitative techniques used to conduct a comprehensive analysis of medical images [10,11]. Applied to CT scans of lung nodules, radiomics is primarily intended to analyse the characteristics of tissues and lesions, with respect to their shape, texture, and intra-tumour heterogeneity, and to monitor changes over time through serial imaging during treatment [12,13]. Examining tissue heterogeneity is crucial, as genomic studies highlight its role as a prognostic factor for survival and a challenge in cancer management [14,15]. Unlike traditional imaging assessment or limited tumour biopsies, radiomics enables heterogeneity assessment across the entire tumour volume. Leveraging large datasets, radiomics identifies previously unrecognised markers and patterns related to disease progression, histological type, and treatment response. Additionally, integrating radiomic data with clinical and pathological datasets through machine learning (ML) enhances the diagnostic and prognostic utility of this approach [16,17].

There are currently no comprehensive systematic reviews evaluating the performance of machine learning (ML)-based radiomic models and DL models specifically aimed at the non-invasive differentiation of lung SCC and ADC. Hence, the aim of this systematic review is to summarise and critically appraise the diagnostic accuracy of artificial intelligence models for the differentiation of SCC and lung ADC.

## 2. Methods

PRISMA (preferred reporting items for systematic reviews and meta-analysis) standards were followed in conducting the review [18] (PRISMA checklist-extended data). The protocol of this review was registered in PROSPERO (CRD420251207481).

### 2.1. Literature Search Strategy

Using databases such as Scopus, PubMed, and Embase, a literature search was conducted to identify suitable original articles (Table 1). Terms including “Lung Carcinoma”, “Lung Cancer”, “artificial intelligence”, “Machine Learning”, “CT Lungs”, and “Non-Small Cell Lung Carcinoma”, along with Boolean operators like “AND” and “OR” (extended data). Only English-language research involving adult subjects who underwent CT examinations was included in the search criteria. Only English-language publications were included to ensure accurate interpretation and the standardised extraction of complex radiomics and machine learning methodological details required for reproducibility assessment and quality scoring.

Mesh terms of three databases is included in Supplementary File S2.

**Selection criteria** A detailed population/patient, intervention, comparator, outcomes (PICO) framework was provided in (Table 2).

### 2.2. Inclusion Criteria

The studies must utilise artificial intelligence models to differentiate between SCC and lung ADC.

Patients included in the study must have pathologically confirmed lung carcinoma, ensuring accurate classification of the histological subtypes.

### 2.3. Exclusion Criteria

Articles that were not original full-text publications, such as reviews, letters, and conference abstracts, were excluded.

Studies that did not provide information specifically on NSCLC classification were excluded.

Articles that did not include radiomics features or machine learning or deep learning approaches were excluded.

### 2.4. Data Extraction

Two researchers independently reviewed each article, extracting details such as the author’s name, publication year, sample size, tumour grade, classifiers used, and study outcomes. Any disagreements between the reviewers were settled by a third researcher.

### 2.5. Quality Assessment

The quality of the studies was assessed using the radiomics quality score (RQS) tool [19]. This tool assesses the ML-based radiomic studies based on criteria with 16 items (underlying data), focusing on methodological rigour, reproducibility, and the clinical relevance of radiomic studies.

## 3. Results

A total of 11 articles were included in the systematic review.

### 3.1. Study Selection

**Figure 1:**
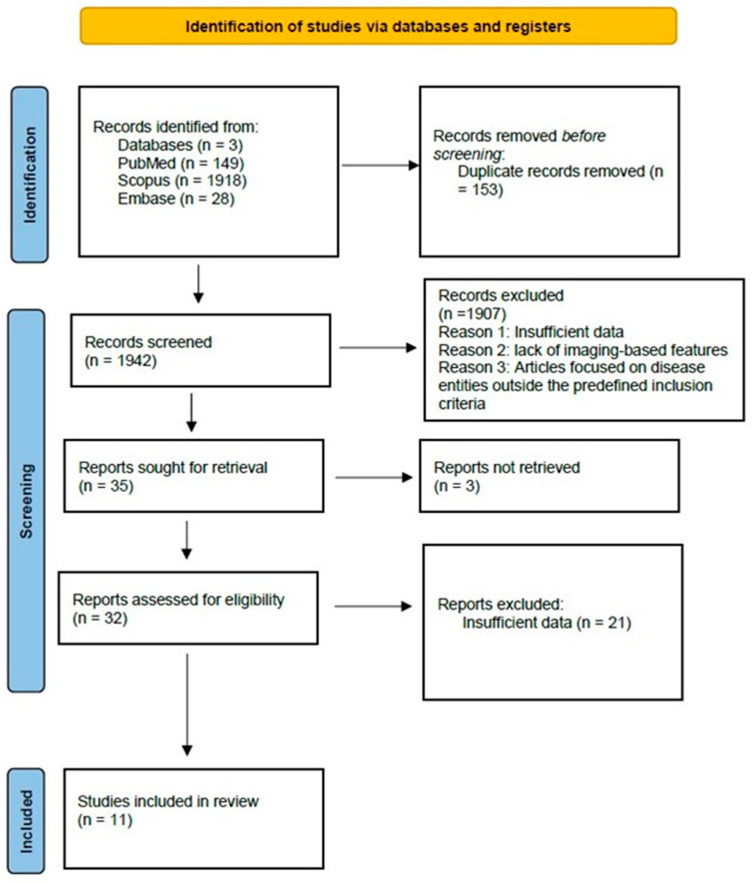
Flow chart for study selection.

According to Figure 1: A total of 2095 studies were found in the early database search. The titles and abstracts of 1942 were examined after 153 duplicates were removed, and 1097 articles that did not fulfil the inclusion criteria were excluded. In the end, 32 articles remained. After evaluating the final text of these 32 articles for eligibility, 21 were disqualified for lacking adequate information, such as a comprehensive table that measured the ML model’s performance. In the end, the final review contained 11 articles.

### 3.2. Interviewer Agreement

The screening and RQS scoring were independently performed by two reviewers following predefined criteria. Any discrepancies noted between the readers were resolved through discussion.

Table 3 shows influence of CT acquisition and segmentation factors on radiomics stability. Radiomics feature stability is strongly influenced by heterogeneity in CT acquisition, reconstruction, and segmentation protocols, as observed across the included studies. Substantial variability was present in scanner vendors and models, ranging from Siemens, GE, Toshiba, Philips, and United Imaging systems to multi-institutional public datasets (TCIA, LUNA16, DeepLesion). Differences in detector configuration and image reconstruction pipelines across vendors are known to alter voxel intensity distributions and texture patterns, thereby affecting feature reproducibility.

Slice thickness showed wide variation (0.7–5 mm), with thinner slices (≈1–1.25 mm) generally offering improved spatial resolution but increased image noise, whereas thicker slices (≥3–5 mm) may smooth fine textural details.

Some studies used non-contrast CT, whereas others employed contrast-enhanced scans with arterial and venous phases. Contrast administration alters lesion attenuation and intratumoral heterogeneity, potentially improving biological signal but reducing feature stability when mixed with non-contrast datasets. Segmentation methodology varied considerably, including single-reader manual delineation, independent multi-observer contouring with consensus correction, and the use of pre-existing dataset annotations. Only a minority of studies explicitly assessed or reported inter-observer agreement.

These findings highlight that scanner heterogeneity, slice thickness variability, reconstruction parameters, contrast usage, and limited inter-observer segmentation assessment represent key methodological limitations in the current literature. Future studies should prioritise standardised acquisition protocols, detailed reporting in accordance with radiomics quality guidelines, and explicit evaluation of feature robustness across observers and scanners to enhance the clinical translation of AI models for differentiating SCC and ADC.

### 3.3. Characteristics of Selected Studies

The review included studies that were conducted across eight countries—China = 4, India = 2, Japan = 1, Brazil = 1, and the United Kingdom = 1, with two articles published in collaboration between South Korea and the Netherlands, and China and the USA—using a variety of CT scanner systems such as GE Healthcare (Lightspeed VCT, Discovery CT750 HD 64-slice), Siemens (SOMATOM Definition Flash dual-source CT), Toshiba (Aquilion LB 16-detector), Philips Gemini TF PET-CT, and United Imaging Healthcare (uCT760), and public databases with multiple scanners, including LUNA16 and TCIA. This extensive geographic representation ensured that the training and validation data encompassed diverse patient populations, healthcare settings, and disease prevalence patterns, which is critical for minimising model bias and enhancing external validity across regions. The total patient cohort was over 4300 cases. Most articles employed a retrospective design, with only a few using prospective or publicly sourced imaging datasets for analysis that aggregate imaging data acquired from multiple institutions and scanner vendors. The study characteristics are shown in Table 4.

### 3.4. Results of RQS Score 

Due to multiple segmentation flaws, the absence of phantom studies, scanning at various times, cutoff analyses, lack of calibration statistics, no prospective data collection, and the absence of a cost-effectiveness analysis, all the studies received scores below 50%. The quality assessment score of the studies included in the review as (Supplementary File S1).

Table 5: Sensitivity analyses demonstrated that the pooled radiomic quality score was robust, with leave-one-out exclusion resulting in minimal variation (maximum change: 0.81%). Item-level analysis revealed consistent reporting of protocol and feature selection components, while reproducibility, validation, and clinical translation items were infrequently addressed.

### 3.5. Imaging Phenotypic Features That Remain Challenging

An analysis of failure modes across the included studies shows consistent patterns of misclassification when differentiating ADC from SCC. Confusion matrix decomposition demonstrated that SCC was more frequently misclassified than ADC, with SCC false-negative rates ranging from approximately 31 to 37% in several studies, whereas ADC false-negative rates were generally lower (8–19%). Multiple studies reported bidirectional confusion between ADC and SCC, indicating substantial phenotypic overlap rather than isolated model bias. SCC consistently emerged as the weakest class in multiclass models, with lower F1 Scores and higher misclassification rates compared to ADC. The dominant failure modes were associated with tumour density overlap, central necrosis/cavitation, central airway location, absence of ground-glass opacity, margin morphology ambiguity, peritumoral tissue similarity, small lesion size (≤5 mm), scanner and protocol variability, and acquisition protocols.

### 3.6. Performance Measures of ML and DL Models for Predicting Non-Small Cell Lung Carcinoma (Table 6)

A study conducted by Rawat et al. [20] for the classification of NSCLC detection using a CT-based deep learning system. The LUNA 16 dataset was used for the initial dataset. The LCDCS system with deep learning performed better against alternative datasets with and without deep learning methods. The deep learning-enabled LCDCS system demonstrates the strongest overall performance in lung cancer diagnosis, with a validation accuracy of 96.9% and training accuracy of 95.8%, outperforming 3D CNN systems, SVM models, and multi-section CNN models, which achieved accuracies of 83.7%, 92%, and 92.17%, respectively. The deep learning model identified 49.9 true positives, 47.0 true negatives, 1.1 false positives, and 2.0 false negatives, yielding a sensitivity of 95.92% and a specificity of 97.85%, which highlights its strong predictive capability.

Gaddala et al. [21] predicted the classification of lung lesions into normal, benign, and malignant, using open-source data from the NIH Clinical Centre and Deep Lesion Collection. Naïve Bayes and Random Forest models were developed, with the Random Forest showing a sensitivity of 94.8%, accuracy of 96.4%, precision of 95.2%, and F1-score of 95%. Naïve Bayes demonstrated a sensitivity of 86.7%, precision of 87.6%, accuracy of 88.5%, and F1-score of 85.4%. The Random Forest outperformed Naïve Bayes in classifying normal, benign, and malignant lung lesions.

Lima et al. [22] used deep learning to predict the classification of NSCLC by histologic subtype. TCIA public datasets were used, and the deep learning model achieved classification accuracies of 84.5% for ADC and 89.6% for SCC, with sensitivities of approximately 91.7% and 90.4%, respectively, and specificities exceeding 99%. The model’s performance in detecting NSCLC subtypes was comparable to or better than previous studies, with high sensitivity and specificity values indicating robust discriminatory power.

Haga et al. [23] predicted and classified histologic subtypes of early-stage NSCLC using radiomic features derived from CT images. Multivariable analysis with Naïve Bayes achieved an average AUC of 0.757 for features selected independently and 0.725 for features that were consistent across different delineations. The original tumour volume delineated by specialists provided the best performance, with an AUC of 0.809. Overall, accuracy, sensitivity, and specificity averaged around 0.70. The study emphasises the significance of multi-delineation analysis for selecting stable radiomic features and indicates that specialist contouring enhances predictive accuracy.

A study by Marentakis et al. [24] focused on predicting the histology of lung cancer classification from CT image-based radiomic features. TCIA datasets were used, and KNN showed the highest accuracy of 68%, with a specificity of 0.68, sensitivity of 0.66, and precision of 0.68. SVM achieved an accuracy of 58%, with a specificity of 0.53, sensitivity of 0.63, and precision of 0.59. A deep learning model was developed using CNN and Inception; the Inception model showed the highest accuracy of 61%, with a sensitivity of 0.65 and specificity of 0.56 when compared with other models. The best-performing models outperformed experts across all evaluation metrics.

In one study, Guo et al. [25] developed ProNet, a 3D deep learning model, and com_radNet, a radiomics model, to classify lung ADC, SCC, and Small Cell Lung Cancer (SCLC) from CT images in 920 patients. ProNet achieved an AUC of 0.840 and an accuracy of 71.6%, with F1-scores of 90.0% (ADC), 72.4% (SCC), and 83.7% (SCLC). com_radNet showed an AUC of 0.789 and an accuracy of 74.7%, with F1-scores of 83.1% (ADC), 75.4% (SCC), and 85.1% (SCLC). Both models effectively distinguished the three cancer subtypes, with ProNet outperforming in overall discrimination, and com_radNet achieving a slightly higher accuracy. The study highlights their potential as non-invasive tools for aiding in the diagnosis of lung cancer subtypes and treatment planning.

A study by Liu et al. [26] showed that the Capsule Network (CapsNet) outperformed the Convolutional Neural Network (CNN) and radiomics-based models in classifying NSCLC subtypes using CT images. CapsNet achieved 81.3% accuracy, 82.2% sensitivity, and 80.7% specificity, with an AUC of 0.852, slightly better than the best radiomics model, Random Forest, which achieved 75.9% accuracy and an AUC of 0.84. CNN achieved 75% accuracy, accompanied by a lower sensitivity of 68.3% and a similar specificity of 80.7%. CapsNet outperformed the radiomics model, demonstrating its potential for accurate NSCLC subtype classification, especially in limited clinical datasets.

Tang et al. [27] employed a radiomics strategy that incorporates intratumoral and peritumoral features extracted from lung CT images using ensemble learning for the pre-treatment prediction of lung SCC and lung ADC. Five machine learning classifiers were combined into an ensemble model: Quadratic Discriminant Analysis (QDA), Support Vector Machine (SVM) with RBF kernel, SVM with sigmoid/tanh kernel, Random Forest (RF), and the training and testing values of AUC for the intratumoral region were 0.87 and 0.66, and, for the peritumoral region, 0.83 and 0.60. The combined intratumoral and peritumoral model achieved AUCs of 0.87 and 0.78, respectively, demonstrating that the combined model outperformed the intratumoral and peritumoral models.

A study by Linning et al. [28] showed radiomics applied to multiphasic contrast-enhanced CT can accurately classify lung cancer histological subtypes. Three radiomics models for classifying ADC versus SCC showed AUCs of 0.801, 0.834, and 0.864, respectively; ADC versus SCLC showed AUCs of 0.857, 0.855, and 0.864; and SCC versus SCLC showed AUCs of 0.657, 0.619, and 0.664, using the plain phase, arterial phase, and venous phase, respectively. Texture features such as Laws_2 and Intensity Entropy were critical predictors. Contrast enhancement affected radiomic feature selection; however, no significant performance difference was found across the different CT phases.

Bashir et al. [29] developed three Random Forest models to classify NSCLC subtypes using semantic features interpreted by radiologists, radiomic features extracted from CT scans, and a combination of both. The radiomic and combined models achieved perfect discrimination with an AUC of 1 on the training data, surpassing the semantic model’s AUC of 0.78 on external validation. The semantic model still maintained strong performance with an AUC of 0.82. Conversely, the radiomic and combined models recorded AUC values of 0.5 and 0.56, respectively.

Saad et al. [30] performed computer-assisted subtyping and prognosis of NSCLC with unresectable tumours and developed a radiomics-based computational method. The GAS Linear 3 and GAS Linear 5 models were created, and the misclassification rate was measured by comparing the predicted subtypes with the actual subtypes for each instance. GAS Linear 3 had a misclassification rate of 3.8% and an accuracy of 96.2%, while GAS Linear 5 had a rate of 17.3% and an accuracy of 82.7% with an AUC of 0.85. Survival analysis was conducted for GAS Linear 3, demonstrating high reproducibility with a concordance correlation coefficient of 0.9910. These results remained consistent between the two methods, although not in T2 and N0 test cases. The overall mean and median survival times for all test cases were identical for both GAS and ICD-O, indicating the reliability of the GAS method.

Independent test set evaluation was performed in a minority of studies, most commonly using external TCIA cohorts from independent institutions.. Studies by Lima et al. [22], Tang et al. [27], Bashir et al. [29] and Saad et al. [30], explicitly reported external validation, whereas the remaining studies relied on internal splits, cross-validation, or out-of-bag estimation.

The best model type and key feature sources were introduced. Deep learning approaches predominantly utilise CNN-based architectures, such as VGG16, 3D CNNs, and CapsNet, extracting features directly from CT images, often after lung masking or tumour segmentation. In contrast, radiomics-based models relied on handcrafted texture, shape, and intensity features, occasionally incorporating peritumoral or multiphasic information, and were commonly paired with classical classifiers such as Random Forests, SVMs, or ensemble methods.

## 4. Discussion

Lung cancer remains the leading cause of cancer-related death worldwide, with NSCLC accounting for nearly 85% of cases. Among its major histological subtypes are lung ADC and SCC. Computed tomography (CT) remains the primary and most widely used imaging modality for detecting, staging, and monitoring lung cancer. Radiomics offers an opportunity to extract a large number of quantitative features from CT images that describe tumour phenotype, heterogeneity, and microenvironment. When combined with machine learning algorithms, these features have shown strong potential for improving the non-invasive classification of ADC and SCC. However, a notable lack of systematic reviews remains in evaluating machine learning- and deep learning-based radiomics models for the classification of NSCLC.

Rawat et al. [20] and Marentakis et al. [24] reported the detection of NSCLC using deep learning based on CT images. Rawat et al. [20] developed a deep learning system using Multi-Level Convolutional Neural Networks (ML-CNN) on CT images, enhanced with data balancing techniques (ADASYN and Tomek Link), achieving a high accuracy (96.9%) for detecting multiple lung cancer types. Marentakis et al. [24] developed a radiomics-based machine learning model and various CNN models combined with LSTM on pre-treatment CT scans. The kNN model achieved an accuracy of 0.67, while the Inception model with LSTM outperformed expert radiologists with an accuracy of 74%. Both studies utilise artificial intelligence (AI) in CT imaging for NSCLC but differ in their foci on detection versus histology classification, data handling approaches, and evaluation metrics, complementing each other in improving lung cancer diagnosis accuracy and clinical utility.

The studies by Linning et al. [28], Guo et al. [25], and Liu et al. [26] aimed to establish non-invasive diagnostic models for lung cancer subtyping using CT imaging and computational analysis. Guo et al. [25] and Liu et al. [26] incorporated deep learning frameworks, such as 3D CNN, ProNet, and CapsNet. Linning et al. developed a machine learning model based on CT radiomic features. Both Guo et al. and Linning et al. [28] included three subtypes (ADC, SCC, and SCLC), whereas Liu et al. limited analysis to ADC versus SCC. Guo et al. [25] developed a 3D deep learning method, which achieved better discrimination with an AUC value of 0.84 compared to the radiomics model, with an AUC value of 0.78. Liu et al. [26] developed CapsNet, which demonstrated higher robustness and automation compared to CNN and radiomics models, with an accuracy of 81.3%, sensitivity of 82.2%, and specificity of 80.7%. However, radiomics using contrast-enhanced phases, as reported by Linning et al. [28], also achieved a comparable AUC with a value of 0.864. Dataset size also influenced performance: Larger datasets, as in those by Guo et al. [25], allowed for stable deep learning training, while CapsNet, as in Liu et al. [26], compensated for smaller datasets with improved spatial representation. Deep learning and radiomics models demonstrated strong potential for accurately and non-invasively classifying lung cancer subtypes, with CapsNet and 3D CNN models offering slightly superior performance compared to traditional radiomics approaches, especially when integrated with multiphasic CT imaging.

Gaddala et al. [21] and Lima et al. [22] both focused on CT-based radiomics and machine learning approaches for differentiating and classifying lung cancer. Gaddala et al. [21] developed a machine learning model using a Random Forest classifier to categorise lung lesions as benign, malignant, or normal, based on CT image features. The model achieved 96.4% accuracy, outperforming a Naïve Bayes classifier, with a precision of 95.2%. In contrast, Lima et al. [22] implemented a deep learning model specifically targeting the histologic differentiation of NSCLC into ADC and SCC. They employed a 2D U-Net for tumour segmentation and a modified VGG16 CNN for classification, with 84.5% accuracy for ADC and 89.6% for SCC, with sensitivities above 90%. Both studies demonstrate that integrating radiomics with machine learning enhances the diagnosis of lung cancer from CT imaging. ML methods achieve high accuracy for general detection, whereas deep learning models enable differentiation between NSCLC subtypes.

Tang et al. [27] and Saad et al. [30] focused on the non-invasive differentiation of lung SCC and ADC using CT imaging. Tang et al. [27] introduced a comprehensive intratumoral and peritumoral radiomics framework, extracting 3078 features and employing a two-step feature selection together with an ensemble of five machine learning classifiers, ultimately achieving an AUC of 0.87 in training and 0.78 in external testing, outperforming a VGG-16 deep network. Saad et al. [30] adopted a 3D co-occurrence-based radiomic feature set and a hybrid Genetic Algorithm–SVM pipeline, achieving a very high subtyping accuracy of 96.2% and demonstrating significant prognostic value through Kaplan–Meier survival stratification, with substantial reproducibility, as indicated by a CCC value of 0.99 when compared to pathological data.

Standardisation of CT image acquisition, reconstruction parameters, and pre-processing techniques is a critical prerequisite for enhancing the robustness, reproducibility, and cross-institutional generalisability of radiomics- and deep learning-based models. Consistent application of comparable scanning protocols, reconstruction kernels, and pre-processing strategies minimises the technical variability that can mask the biologically relevant imaging features.

Future studies should explicitly distinguish between AI models developed for pulmonary nodule or lesion detection and those designed for histological subtype classification, with separate reporting of performance metrics, datasets, and validation strategies.

This review has several limitations. First, a meta-analysis could not be performed due to substantial heterogeneity in the included studies, particularly variations in CT acquisition protocols, radiomics feature extraction methods, and patient cohorts. Second, many studies were based on relatively small sample sizes, which may restrict the generalisability. Third, the predominance of retrospective research designs introduces a risk of selection bias. All included studies, including those evaluating high-performing deep learning models, did not report the use of model interpretability or explainability techniques and were, therefore, subject to inherent black-box limitations. Meta-analysis was not performed because the included studies demonstrated substantial methodological heterogeneity across imaging protocols, AI model architectures, feature extraction pipelines, outcome definitions, and validation strategies.

## 5. Conclusions

This systematic review demonstrates that deep learning and radiomics-driven machine learning models substantially improve the non-invasive and accurate differentiation of lung SCC and ADC when integrated with clinical and histopathological information. Deep learning techniques consistently outperformed machine learning models, achieving superior accuracy, sensitivity, and specificity in detecting and classifying SCC and ADC. Systems such as LCDCS, ProNet, CapsNet, and ensemble radiomics frameworks showed remarkable diagnostic performance.

Radiomics-based models also proved valuable, especially when combined with expert-guided tumour delineation and multiphasic contrast-enhanced imaging, contributing to improved stability and subtype prediction accuracy. Despite the variability in datasets, imaging protocols, and validation strategies, findings collectively highlight deep learning and radiomics-driven machine learning models potential to diagnose squamous cell carcinoma and adenocarcinoma non-invasively, for early detection, and for precise tumour characterisation.

## Figures and Tables

**Table 1:** Study retrieval method from database.

Database	Number of Studies Retrieved	Total
PubMed	149	2095
Scopus	1918	
Embase	28	

**Table 2:** PICO terms.

Population	Patients with Pathologically Confirmed Squamous Cell Carcinoma and Adenocarcinoma
Intervention	Computed tomography of lungs, artificial intelligence
Comparator	Histopathological diagnosis is the gold standard for the diagnosis of Squamous Cell Carcinoma (SCC) and Adenocarcinoma of the lung (ADC)
Outcome	Diagnostic performance measures such as Accuracy, Sensitivity, Specificity, Area Under Curve, F1-score, Precision, Recall

**Table 3:** Showing summary of CT Scanner Models, Acquisition Settings, Contrast Phase and Segmentation Methodology.

Author	CT Machine/Database	Slice Thickness	Kernel Function	Contrast Agent	Phase	Segmentation Software	Interobserver Segmentation Agreement
Rawat et al. [20].	Open source data LUNA 16	Not reported	Not reported	Not reported	Not reported	Not reported	Not reported
Gadalla et al. [21].	Open source data-NIH deeplesion	Not reported	Not reported	Not reported	Not reported	Not reported	Not reported
Lima et al. [22].	Philips Gemini TF PET CT And Open source data TCIA and IMCA dataset	1 mm	Not reported	Not reported	Not reported	2D U-Net archtecture	Not reported
Haga et al. [23].	Toshiba Aquilion LB 16 detector	2 mm	Not reported	Not reported	Not reported	Pinnacle Treatment Planning System, version 9.10 (Philips)	Assessed via multi-observer VOI comparison
Marentakis et al. [24].	NSCLC radiomics dataset-TCIA	3 mm	Not reported	Not reported	Not reported	GTV delineations in the TCIA dataset.	Not reported
Guo et al. [25].	United Imaging 128 slice uCT760 and GE lightspeed VCT 64 slice scanner	1.5 and 1.25 mm	Not reported	Non-contrast	Non-contrast	ITK-SNAP 3.6.0	Not reported
Liu et al. [26].	GE Discovery CT 750 HD 64 slice scanner	1.25 mm	Not reported	Non-contrast	Non-contrast	ITK-SNAP 3.4.0 PyRadiomics 1.2.0	Consensus discussion and manual modification
Tang et al. [27].	United Imaging uCT 760	5 mm	Not reported	Not reported	Not reported	PyRadiomics (version 3.0.1)	Independent delineation followed by consensus correction
Linning et al. [28].	Siemens Somatom Defination flash dual source CT scanner	1 mm	B30f	Omnipaque 350	Arterial and venous	Weasis 4.3.0	A single radiologist only
Bashir et al. [29].	Philips MX8000; Philips Brilliance iCT 256; Philips Brilliance 40	0.75 mm	Not reported	Iopromide 300	30–70 s delay	ITK-SNAP 3.4.0	Not reported
Saad et al. [30].	Not reported	0.7 to 3 mm	Not reported	Not reported	Not reported	MATLAB (R2015b)	Not reported

**Table 4:** Showing the characteristics of selected studies.

SI No	Author and Year	Purpose	Sample Size	Classifiers Used	Outcome of the study
1	Rawat et al. [20].	Classify lung nodules into categories such as benign tissue, Large Cell Carcinoma, and SCC.	1000+ images	Multi-layer Convolutional Neural Network (ML-CNN), which integrates the outputs of four CNNs. The SoftMax function is applied for multi-class classification.	Training accuracy: 95.8%. Validation accuracy: 96.9%.
2	Gaddala et al. [21].	To classify lung cancer as benign, malignant, or normal using a machine learning-based method for improved accuracy in lung cancer diagnosis on CT scans.	4400 images	The primary classifier used is the Random Forest algorithm. Linear Discriminant Analysis (LDA) is used for feature reduction.	The suggested model’s accuracy on CT scans is increased by utilising the Random Forest algorithm for detection. The performance is evaluated using metrics such as accuracy, precision, sensitivity, and recall.
3	Lima et al. [22].	To develop a deep learning system for the classification of SCC and ADC from segmented computed tomography images.	3080 images	1. Segmentation: 2D U-Net neural network. 2. Classification: VGG16-based network, modified for better performance, utilising transfer learning.	The system achieved satisfactory accuracy in detecting NSCLC subtypes.
4	Haga et al. [23].	To evaluate the application of radiomics for predicting the histology of early-stage Non-Small Cell Lung Cancer (NSCLC).	40 patients	A Naïve Bayes model was employed as the multivariable classifier.	The highest AUC averaged over all VOIs was 0.757 ± 0.035 using the eight top-ranked non-correlated features selected without interobserver delineation analysis. Using features selected based on interobserver delineation analysis, the VOI-averaged AUC was 0.725 ± 0.070 for the eight top-ranked non-correlated features.
5	Marentakis et al. [24].	To investigate the potential of NSCLC histology classification, SCC, and ADC.	102 patients	Radiomics: kNN and SVM. Deep learning: Four pre-trained CNNs, AlexNet, ResNet101, Inceptionv3, and InceptionResnetv2, using transfer learning and fine-tuning. Recurrent neural network: LSTM combined with the best-performing CNN (LSTM + Inception). 4. Combinatorial models (LSTM+ CNN+ radiomics).	The LSTM + Inception model yielded the best performance, demonstrating superior results compared to all other methods, including those of expert radiologists.
6	Guo et al. [25].	To automatically distinguish between three histological subtypes of lung cancer, SCC, ADC, and Small Cell Lung Cancer (SCLC).	920 patients	1. ProNet: A self-developed 3D Convolutional Network based on Res-Net architecture. 2. com_radNet: A fully connected network utilising 20 selected radiomic features extracted from delineated tumour regions.	ProNet: AUC = 0.840; Accuracy = 71.6%. com_radNet: AUC = 0.789; Accuracy = 74.7%. The ProNet model showed better AUC, while the com_radNet model showed higher accuracy.
7	Liu et al. [26].	To develop an automated model for discriminating between the subtypes of SCC and ADC.	126 patients	1. CapsNet: Capsule Net model. 2. CNN: A comparative Convolutional Neural Network. 3. Radiomics: Used 107 radiomic features plus six clinical/demographic features with four traditional machine learning classifiers: Random Forest (RF), Logistic Regression (LR), LR with L1 regularisation (LR-L1), and LR with PCA (LR-PCA).	CapsNet achieved the best discriminative performance among all models, demonstrating its potential for limited single-centre datasets.
8	Tang et al. [27].	To evaluate a new radiomics strategy for intratumoral and peritumoral features extracted from lung CT images with ensemble learning for the pretreatment prediction of SCC and ADC.	105 patients	The primary model was an Ensemble classifier combining five machine learning classifiers.	The ensemble classifier achieved the best performance in distinguishing LUSC from LUAD.
9	Linning E. et al. [28].	To evaluate the performance of the radiomics method in classifying the histological subtypes of lung cancer, SCC, ADC, and SCLC.	229 patients	Radiomics approach using 1160 quantitative image features (869 reproducible features). Optimal classification models were built using three machine learning algorithms: Naïve Bayes, linear logistic regression, and Random Forest.	Radiomics showed high performance in differentiating AD vs. SCC: Highest AUC of 0.864 (at venous phase). AD vs. SCLC: Highest AUC of 0.864 (at venous phase). The model showed weak performance in classifying SCC vs. SCLC: Highest AUC of 0.664 (at venous phase).
10	Bashir U. et al. [29].	To compare the performances of Random Forest (RF) algorithms.	106 patients	Random Forest (RF) models: RF-sem (semantic features only). RF-rad (radiomics features only). RF-all (combined semantic and radiomics features).	The RF-sem model demonstrated the best and most consistent performance on the independent test data: RF-sem Test AUC: 0.82. RF-rad Test AUC: 0.52 RF-all Test AUC: 0.56.
Overall Survival Analysis					
11	Saad et al. [30].	To propose a computational method that fuses subtyping and prognosis to non-invasively classify SCC and ADC.	82 patients	A hybrid machine learning technique called GAS (Genetic Algorithms and Support Vector Machines—SVMs) was used. The optimal model was identified as GAS Linear 3 (SVM with linear kernel combined with GA for feature selection).	The computational method demonstrated high accuracy in subtyping and good consistency in prognosis prediction compared to pathological subtyping.

**Table 5:** Sensitivity analysis of RQS.

Study	Mean RQS (%) After Exclusion	Change from Overall Mean (%)
Rawat et al. [20]	39.39	+0.30
Gaddala et al. [21]	39.17	+0.07
Lima et al. [22]	38.84	−0.26
Haga et al. [23]	38.28	−0.81
Marentakis et al. [24]	38.84	−0.26
Guo et al. [25]	39.39	+0.30
Liu et al. [26]	39.67	+0.58
Tang et al. [27]	39.39	+0.30
Linning et al. [28]	38.84	−0.26
Bashir et al. [29]	39.67	+0.58
Saad et al. [30]	38.56	−0.53

**Table 6:** Showing the performance measures of various models across different studies.

Author (Year)	Task/Outcome	Dataset Split	Evaluation Type	AUC	Sensitivity	Specificity	Accuracy/F1	Best Model Type	Key Feature Sources
Rawat et al. [20]	NSCLC detection and classification	Train/Validation	Internal validation	NR	95.9%	98.0%	Acc: 96.9%; F1: 96.8%	Deep learning-enabled LCDCS	CNN-derived multi-scale image features
Gaddala et al. [21]	Lung cancer detection (normal vs. benign vs. malignant)	Train/Test	Internal test set	NR	94.8%	NR	Acc: 96.4%; F1: 95.0%	Random Forest	Handcrafted radiomic texture and intensity features
Lima et al. [22]	NSCLC detection and histologic subtype classification (ADC vs. SCC)	Training: TCIA; Validation/Test: INCA	Independent external test set	NR	91.7% (ADC), 90.4% (SCC)	99.3% (ADC), 99.5% (SCC)	Acc: 84.5% (ADC), 89.6% (SCC)	VGG16-based DL model (fine-tuned)	Deep CNN features from lung-masked CT images
Haga et al. [23]	Early-stage NSCLC histology classification	Train/Test (random sampling, repeated)	Internal test set	0.809 (best VOI)	NR	NR	Acc, Sen, Spec reported (VOI-averaged)	Naïve Bayes	Radiomic features from multiple VOIs
Marentakis et al. [24]	NSCLC histological subtype classification	Train/Test	Internal test set	0.78	0.81	0.67	Acc: 0.74; F1: NR	LSTM + Inception CNN	Deep spatiotemporal CNN features
Marentakis et al. [24]	NSCLC histology classification	Cross-validation	Internal validation	NR	0.66–0.74	0.53–0.68	Acc: 0.58–0.70	kNN (radiomics); Inception (CNN)	Handcrafted radiomics; CNN deep features
Guo et al. [25]	Lung cancer histological subtype classification (ADC, SCC, SCLC)	Train/Validation/Test (internal split)	Internal validation + internal test	0.840 (DL), 0.789 (Radiomics)	NR	NR	Acc: 71.6% (DL), 74.7% (radiomics); F1 (weighted): 73.2%	3D CNN (ProNet)	3D CT deep features ± selected radiomics
Liu et al. [26]	AC vs. SCC	Internal cross-validation	Internal	NR	82.2	80.7	81.3	Capsule Network (CapsNet)	Manually segmented intratumoral CT features; deep capsule representations
Tang et al. [27]	LUAD vs. LUSC	Training cohort (n = 73)	Internal	0.87	NR	NR	NR	Ensemble radiomics classifier	Intra- and peritumoral radiomics (shape, first-order, texture)
Tang et al. [27]	LUAD vs. LUSC	Independent test cohort (n = 32)	Independent	0.78	NR	NR	NR	Ensemble radiomics classifier	Combined intra- and peritumoral CT radiomics
Linning et al. [28]	AD vs. SCC	Non-enhanced CT	Internal	0.801	NR	NR	NR	Radiomics + RF/LR/NB	Shape, texture, intensity features
Linning et al. [28]	AD vs. SCC	Arterial phase CT	Internal	0.834	NR	NR	NR	Radiomics + RF/LR/NB	Multiphasic contrast-enhanced radiomics
Linning et al. [28]	AD vs. SCC	Venous phase CT	Internal	0.864	NR	NR	NR	Radiomics + RF/LR/NB	Multiphasic contrast-enhanced radiomics
Bashir et al. [29]	AD vs. SCC	Training (internal OOB)	Internal	1.00	NR	NR	NR	RF-radiomics/RF-combined	Radiomics ± semantic CT features
Bashir et al. [29]	AD vs. SCC	Independent TCIA test set	Independent	0.82	NR	NR	NR	RF-semantic features	Radiologist-derived semantic CT descriptors
Saad et al. [30]	ADC vs. SCC	Testing (Lung1)	Independent	NR	NR	NR	NR	GA-SVM hybrid radiomics model	Same as training; independent TCIA cohort

## Data Availability

No new data were created or analyzed in this study.

## Supplementary Materials

The following supporting information can be downloaded at: https://www.mdpi.com/article/10.3390/diagnostics16030500/s1, Supplementary File S1: Radiomic quality scores of studies and Supplementary File S2: MeSH Terms used for databases.

## Abbreviations

The following abbreviations are used in this manuscript:

NSCLC	Non-Small Cell Lung Carcinoma
SCC	Squamous Cell Carcinoma
ADC	Adenocarcinoma
CT	Computed tomography
ML	Machine learning
PRISMA	Preferred reporting items for systematic review and meta-analysis
RQS	Radiomic quality score
CNN	Convolutional Neural Network
LDA	Linear data analysis
AUC	Area under curve
VOI	Volume of interest
VGG	Visual geometry group
KNN	k-Nearest Neighbours
LSTM	Long short-term memory
RF	Random Forests
LR	Logistic regression
PCA	Principal component analysis
LUSC	Lung Squamous Cell Carcinoma
LUAD	Lung Adenocarcinoma
GAS	Genetic Algorithms
SVM	Support Vector Machine
SMOTE	Synthetic memory over sampling techniques
QDA	Quadratic discriminant analysis
ICD	Interface central document
ADASYN	Adaptive synthetic sampling
SCLC	Small Cell Lung Carcinoma
CCC	Concordance correlation coefficient
