# Supplementary material for: Diagnostic Accuracy of Artificial Intelligence Models for Differentiation of Squamous Cell Carcinoma and Adenocarcinoma of Lung—A Systematic Review
Source: Diagnostics (Basel). 2026 Feb 6;16(3):500. doi: 10.3390/diagnostics16030500 (PMC12896415; doi:10.3390/diagnostics16030500)
Supplement: Supplementary file 1 [file diagnostics-16-00500-s001.zip › diagnostics-4079760-supplementary.pdf]

Supplementary File S1: Radiomic quality scores of studies

Study and year	Item 1	Item 2	Item 3	Item 4	Item 5	Item 6	Item 7	Item 8	Item 9	Item 10	Item 11	Item 12	Item 13	Item 14	Item 15	Item 16	RQS Percentage
Rawat et al [20]	1	1	0	0	2	1	0	0	0	0	0	1	1	1	0	1	36.11%
Gaddala et al [21]	1	1	0	0	2	0	0	1	1	0	0	1	1	1	0	1	38.39%
Lima et al [22]	1	0	0	0	2	0	1	0	0	1	1	1	1	1	0	1	41.67%
Haga et al [23]	1	1	0	0	2	0	1	0	2	0	0	1	1	1	0	1	47.22%
Marentakis et al [24]	1	0	0	0	2	1	1	0	2	0	0	1	1	1	0	1	41.67%
Guo et al [25]	1	1	0	0	2	0	0	0	2	1	0	1	1	0	0	1	36.11%
Liu et al [26]	1	0	0	0	2	1	0	0	2	0	0	1	0	1	0	1	33.33%
Tang et al [27]	1	1	0	0	2	0	0	0	1	0	0	1	1	1	0	1	36.11%
Linning E et al [28]	1	0	0	0	2	0	1	1	2	0	0	1	1	1	0	1	41.67%
Bashir et al [29]	1	0	0	0	2	0	1	1	1	0	0	1	1	0	0	1	33.33%
Saad et al [30]	1	0	0	0	2	0	1	1	1	1	0	1	1	1	0	1	44.44%

Supplementary File S2: MeSH terms used for databases

PubMed

"((((((""lung""[MeSH Terms] OR ""lung""[All Fields]) AND (""carcinoma""[MeSH Terms] OR ""carcinoma""[All Fields] OR ""carcinomas""[All Fields] OR ""carcinoma s""[All Fields])) OR (""lung neoplasms""[MeSH Terms] OR (""lung""[All Fields] AND ""neoplasms""[All Fields]) OR ""lung neoplasms""[All Fields] OR (""lung""[All Fields] AND ""cancer""[All Fields]) OR ""lung cancer""[All Fields])) AND (""artificial intelligence""[MeSH Terms] OR (""artificial""[All Fields] AND ""intelligence""[All Fields]) OR ""artificial intelligence""[All Fields])) OR (""machine learning""[MeSH Terms] OR (""machine""[All Fields] AND ""learning""[All Fields]) OR ""machine learning""[All Fields])) AND ((""j comput tomogr""[Journal] OR ""commun theory""[Journal] OR ""child teenagers""[Journal] OR ""cancer ther""[Journal] OR ""ct""[All Fields]) AND (""lung""[MeSH Terms] OR ""lung""[All Fields] OR ""lungs""[All Fields] OR ""lung s""[All Fields])) AND (""carcinoma””)

Scopus

Your query : (lung carcinoma OR lung cancer AND artificial intelligence OR machine learning AND ct lungs AND Non-Small Cell Lung Carcinoma AND PUBYEAR > 2014 AND PUBYEAR < 2026 AND (LIMIT-TO (SUBJAREA,"MEDI")) AND (LIMIT-TO (DOCTYPE,"ar")) AND (LIMIT-TO (EXACTKEYWORD,"Human") OR LIMIT-TO (EXACTKEYWORD,"Article")) AND (LIMIT-TO (LANGUAGE,"English")) AND (LIMIT-TO (PUBSTAGE,"final")))

Embase

((('lung'/exp OR lung) AND ('carcinoma'/exp OR carcinoma) OR (('lung'/exp OR lung) AND ('cancer'/exp OR cancer))) AND artificial AND ('intelligence'/exp OR intelligence) OR (('machine'/exp OR machine) AND ('learning'/exp OR learning))) AND ('ct'/exp OR ct) AND ('lungs'/exp OR lungs) AND 'non small' AND ('cell'/exp OR cell) AND ('lung'/exp OR lung) AND ('carcinoma'/exp OR carcinoma)
